# Influence of Hulling, Cleaning and Brushing/Polishing of (Pseudo)Cereal Grains on Compositional Characteristics

**DOI:** 10.3390/foods12132452

**Published:** 2023-06-22

**Authors:** Lovro Sinkovič, Barbara Pipan, Mohamed Neji, Marianna Rakszegi, Vladimir Meglič

**Affiliations:** 1 Crop Science Department, Agricultural Institute of Slovenia, Hacquetova ulica 17, SI-1000 Ljubljana, Slovenia; barbara.pipan@kis.si (B.P.); mohamed.neji@kis.si (M.N.); vladimir.meglic@kis.si (V.M.); 2 Cereal Breeding Department, Agricultural Institute, Centre for Agricultural Research, Brunszvik u. 2, 2462 Martonvásár, Hungary; rakszegi.mariann@atk.hu

**Keywords:** cereals, pseudocereals, grain fraction, elements, ICP-MS, β-glucan, protein

## Abstract

(Pseudo)cereal grains have been the basis of human nutrition for thousands of years. The various types of cereals are usually harvested by grain harvesters and must be technologically processed in different ways before consumption. In addition to genotype and growing conditions, the compositional characteristics of the (pseudo)cereal grains are highly dependent on the processes used. In the present study, the effects of hulling, cleaning and brushing/polishing wheat, spelt, oat, barley, common and Tartary buckwheat grains and their fractions on physical parameters (thousand kernel weight, kernel width, fractional yield) and nutritional characteristics (protein, fat, β-glucan, macro- and microelements) were investigated. Grain samples contained 22.7–148.5 mg/g protein, 4.5–69.6 mg/g fat and 0.5–54.4 mg/g β-glucan. The content of macro- (K, Mg, P, S, Ca) and microelements (Mn, Fe, Zn, Na, Cu, Cr, Mo) varied considerably among the studied (pseudo)cereals and their grain fractions. Analysis of variance showed that species and fractions significantly influenced most of the analyzed characteristics. However, the composition of the edible fractions was not significantly dependent on the brushing/polishing process.

## 1. Introduction

(Pseudo)cereals are the most important source of energy in the human diet. In addition to high carbohydrate content (70–80%), they also have high protein (7.5–15%) and mineral content (1.5–3%) and low fat content (1–4%) [1,2,3]. However, the chemical composition of different cereals varies widely due to different genetic background, various environmental and agrotechnical factors and their interactions [4]. Grain processing is required for virtually all cereals that humans consume, transforming them into palatable, nutritious and convenient whole-grain food products that are higher in dietary fiber and associated nutrients and phytochemicals than refined-grain comparators [5].

The whole grain fractions of barley, oat, wheat, spelt and buckwheat genotypes have an excellent composition of nutrients and bioactive components due to the presence of bran and germ [6]. The groats produced during hulling are also whole grains containing the germ, fiber-rich bran part and endosperm [7,8]. It is therefore not surprising that nutrition experts increasingly recommend the consumption of less processed cereal grain products [1]. Cereal products made from cleaned, hulled and brushed/polished wheat, spelt, barley, oat and buckwheat grains can be used as ingredients in the preparation of traditional and modern dishes. Examples of such whole grain foods are wheat groats, spelt rice, spelt groats, spelt kasha, barley groats, barley kasha, pot barley, barley porridge, barley pearls, whole oat groats, whole grain oats, steel cut oats, oat rice or a mixture of different grains (rice, spelt and barley, etc.) [9,10,11,12].

In order to produce these foods, the “raw” harvested grains must be cleaned and brushed/polished through a special technological process before they can be consumed by humans. First, the chaff is separated from the grain by threshing; then, the seed is cleaned from other impurities, including pests, by winnowing [13]. Further processing of the grains pre-cleaned grain involves fine brushing/polishing, which removes dust, spores, fungal and contaminant particles adhering to the grains without affecting germinating capacity. During polishing, a part of the grain is lost; the amount of loss depends on the cereal type, the duration of the treatment and/or the number of times the operation is repeated. Although the removal of dust and impurities from the grain is beneficial to health, some of the bioactive components have also been removed. The loss of these constituents and the compositional properties of the polished fractions have been studied in only a limited number of publications.

Wheat (*Triticum aestivum* L.) accounts for 20% of daily energy intake and about 19% of daily protein intake worldwide [14]. In addition, wheat is one of the main sources of dietary fiber and one of the most important sources for the intake of other essential micronutrients such as iron (Fe), zinc (Zn) or B-complex vitamins. These nutrients accumulate in varying concentrations in different parts of the wheat grain. For example, the endosperm, which accounts for most of the grain mass, is the main starch and protein store in the wheat grain, while the bran, aleurone and germ are rich in vitamins, minerals, polyphenols and fats [15]. The protein content in wheat grains can range from 6.2 to 19.8% and is usually higher in hard wheats such as spelt [16].

Spelt (*Triticum spelta* L.) is an ancient hulled wheat that has attracted new interest in recent years because it is a low-input crop suitable for pesticide-free cultivation in organic farming [17]. The disadvantage of spelt cultivation is the lodging of the plants and the need to hull the grains after harvest [18]. Spelt grains are covered with a strong chaff, which protects them from external harmful influences but makes harvesting and processing difficult. Therefore, the grains need additional threshing after harvesting spelt wheat [19]. The grain composition of spelt wheat is essentially similar to that of wheat, but higher protein and alkylresorcin content and lower fructan content were found compared to wheat [20,21].

Barley grains (*Hordeum vulgare* L.) are mainly composed of starch (50–65%), while proteins constitute 10–16% of barley grains and fiber 13–22%. Besides these main components, barley is a source of vitamins (especially vitamin E, tocopherols) and microelements (Fe and Zn). The storage proteins of barley do not contain sufficient essential amino acids, especially lysine, but the β-glucan content of barley is exceptionally high (2.5–11.5%), which makes it ideal for the production of functional foods [15,22]. Minerals are found mainly in the seed coat, aleurone and germ (21–83 mg/kg Fe and 6–38 mg/kg Zn). High variability and generally higher Fe and Zn concentrations were found between barley landraces from Ethiopia and Eritrea and wild barley species. Very high variability was also found in barley phytic acid concentrations. Barley varieties with colored seeds are rich in anthocyanins, proanthocyanins and phytomelatonins, as well as other health-promoting compounds. Thus, blue and black barley contain more phenolic compounds compared to white barley [15].

Among cereals, oats (*Avena sativa* L.) are particularly rich in proteins (globulins), phenolic compounds and dietary fibers, especially β-glucan, as well as various vitamins and minerals [4]. In addition, oats are rich in fat and thiamine content, while their energy value is higher than that of other cereals [3]. The highest quantity of proteins is found in the germ (over 30%), followed by the aleurone layer and bran (20–25%), while the least amount is found in the endosperm (10–15%). ß-glucan is concentrated in the subaleuron layer and in the cell wall of the endosperm. Among cereals, oats have the highest fat content (2–11%) and a unique composition of antioxidants. Besides vitamin E, it contains avenanthramides (unique to oats), phenolic acids, flavonoids, sterols and phytic acid. Most minerals in oat grains (≈70%) are bound to soluble fibers (>50% Ca, Fe, Mn and P), possibly β-glucans and/or phytates [23].

Pseudocereals, such as common and Tartary buckwheat, are non-grass wild plants whose grains are used in the same way as cereals but are underutilized due to the dominance of conventional cereal crops [24]. Common buckwheat is the most well-known buckwheat species due to its pleasant taste, large seed size and easy-to-hull seed coat. Tartary buckwheat species has a bitter taste, smaller seed size and a dense seed coat that makes it difficult to hull, making it is less popular with growers and consumers. Buckwheat protein contains a large amount of the amino acid lysine, which is usually the limiting amino acid of plant proteins [25]. The protein content of common and Tartary buckwheat is ≈12% and can be concentrated by milling and separation of fractions [26]. The fat content is highest in the bran fraction (9–20%), while in the endosperm it is only 2–3%. Buckwheat is a source of flavonoid (rutin) for the production of herbal medicines. Rutin content in dark buckwheat flour is higher than in raw (uncooked) groats. Dietary fiber is mainly found in the seed coat and husk of the buckwheat grain. Similar to oats and wheat, buckwheat groats contain much less dietary fiber (≈7%) in than the grain. Buckwheat also contains a significant quantity of lignans and unique carbohydrate molecules known as fagopyritols, which are concentrated mainly in the embryo and aleurone tissues. Buckwheat has the highest concentration of these sugars among plant sources [25]. Buckwheat groats can be produced in different ways, as husking can be conducted via non-precooked or precooked grains [27].

The aim of our study was to determine and compare the physical parameters and grain composition of four cereals (wheat, spelt, barley, oat) and two pseudocereals (common buckwheat, Tartary buckwheat) and their grain fractions. The influence of minimal processing was investigated, in which harvested, air-dried grain samples were processed using various non-thermal techniques or combinations thereof (e.g., threshing, winnowing, brushing/polishing, hulling). Grain processing tests on different (pseudo)cereals are crucial for the development of processing equipment and new nutrient-rich wholegrain cereal foods.

## 2. Materials and Methods

### 2.1. Plant Materials and Grain Processing

Six (pseudo)cereals (Table 1), i.e., wheat (CCB Ingenio), spelt (Murska bela), oat (Noni), barley (Concordia), common buckwheat (Eva) and Tartary buckwheat (Doris), were grown according to the management systems established for each species on the experimental fields of the Infrastructural Centre Jablje at the Agricultural Institute of Slovenia (46°30′17.4″ N, 15°37′34.6″ E; 320 m a.s.l., subalpine climate) during the winter and/or spring growing seasons of 2017–2018. CCB Ingenio is an early winter wheat variety with high yield potential and very good grain quality. A standard quality B1 variety with excellent protein content under optimal production conditions. Plants are of medium height and tolerant to lodging, *Septoria* and *Fusarium*. Stability and adaptability under different agroecological conditions and on different soil types is high; moreover, it is the recommended winter wheat variety for cultivation on medium deep soils in Slovenia. Murska Bela is a conservation winter variety of spelt registered in the Slovenian National List of Varieties. The variety was produced by individual selection from the bulk population and is one of the most widespread varieties in Slovenian cultivation. Awns and scurs are absent, while the ear color is white. Concordia is a two-row winter barley variety that is widely grown in Slovenia and is a standard variety for buyers and millers. It is a medium-late variety characterized by a very large ear and very good grain uniformity. Concordia has been a winner of grain yield in many demonstration trials for many years. Noni is a spring type and a protected oat variety on the Slovenian National List of Varieties. Plants are tall, grain is medium in size and light ochre in color. The variety matures medium-late and is resistant to diseases and lodging. Protein content is high, and yields are very good. Eva is a conserved common buckwheat variety that is on the Slovenian National List of Varieties. Compared to established buckwheat varieties, this variety occurs in cultivation at a very low level, so there is a high probability that without seed production and conservation selection, it will be lost and not cultivated or its genetic potential for cultivation will be lost due to genetic erosion. The variety is adapted to poorer growing conditions and is suitable for cultivation in organic farms. It is resistant to diseases, belongs to early varieties, has white flowers and a high habitus. The grain is dark brown and is suitable for processing into buckwheat flour. Doris is in Distinctness, Uniformity and Stability (DUS) testing as a promising candidate for a new conservation variety of Tartary buckwheat. It is naturally adapted to local and regional conditions and threatened by genetic erosion and is grown on a very small scale in Slovenia. The variety is suitable for cultivation on organic land/farm. Plants bloom late, have medium-to-tall growth habit with a large number of nodes on the stem and are disease resistant. The flowers are light green or slightly reddish and belong to the late varieties. The grain has a light or dark brown color and is suitable for processing into both flour and kasha.

The harvested grains were air-dried and about 30 kg of a representative grain sample of each (pseudo)cereal type was stored until further processing. Wheat, barley, oat, common and Tartary buckwheat grains were cleaned using a grain winnowing machine with a capacity of 100 kg/h. For spelt grains, an additional step was required, namely, threshing with the Wintersteiger LD350 (Wintersteiger AB, Arnstadt, Germany), while the Haldrup DC-20 densimetric column (Haldrup GmbH, Ilshofen, Germany) was used for cleaning. Pre-cleaned grains (wheat), grains with husks (barley, oats) or hulled grains (spelt) were further processed by brushing/polishing (wheat 3×, barley 3×, oats 6×, spelt 3×), based on centrifugal force in a stone mill with a series of sieves specially adapted for processing cereal grains. This simple brushing polishes and removes dust, spores, fungal and dirt particles adhering to the grains without affecting their germination capacity. The four grain fractions for wheat, spelt, barley and oat consisted of least polished, medium polished, most polished and husks. The pre-cleaned grains of common and Tartary buckwheat were hulled using the “Sheller” device with dimensions 1.5/1.5/2.0 m, hulling capacity 20–30 kg/h, motor power 1100 W and voltage 380 V (Craft grain mills, sieves and shellers—MPP d.o.o., Brezje, Slovenia). The total of 25 grain samples used for further composition characterisation consisted of “raw” harvested/unpolished grains and their grain fractions; that is, least-, medium- and most-polished grains and husks.

### 2.2. Physical and Nutritional Analyses

The thousand kernel weight (TKW; g) and kernel width (mm) parameters of the grain samples were determined using the Marvin system (MarviTech GmbH, Wittenburg, Germany) where applicable. Yield of each grain fraction for individual (pseudo)cereal type was calculated as a % based on whole grains as 100% (Table 2). All grain samples were then homogenized in a laboratory ball mill (Retsch MM400, Retsch GmbH, Haan, Germany) prior to further nutrient and elemental analysis. Dry matter content was determined by drying the samples at 130 °C for 1 h, cooling in a desiccator and determining the tare weight (EC 152/2009 App. III A, Brussels, Belgium). The Kjeldahl method (ISO 5983:2, 2009) with a factor of 6.25 was used to determine crude protein content. Crude fats were analyzed by petroleum ether extraction (152/2009 App. III H). The content of mixed linkage β-glucan was measured in the homogenized samples by enzymatic digestion and spectrophotometry (Evolution 60S, Thermo Fisher Scientific, Waltham, MA, USA) according to AACC 32–23.01. The measurements were performed in two replicates. Results of protein, fat and β-glucan contents are given on a dry matter basis and are expressed in mg/g.

### 2.3. Elemental Composition

Multielement analysis (for Na, Mg, P, S, K, Ca, Cr, Mn, Fe, Cu, Zn, Mo) was performed using inductively coupled plasma mass spectrometry (ICP-MS). Homogenized grain samples (250 mg) were mixed with 6.0 mL of 65% (*v*/*v*) nitric acid (Suprapur, Merck, Germany) and 2 mL of 30% (*v*/*v*) hydrogen peroxide (Suprapur, Merck) and digested using a microwave-based digestion system (Ethos UP). The digested solutions were diluted to 50 mL with double-deionized water. Elements in the samples were determined using an octopole reaction system (ICP-MS 7900; Agilent, Tokyo, Japan). He was used as the reaction gas at a flow rate of 5.0 mL/min in He mode and 10.0 mL/min in HEHe mode. The calibration curve was prepared using a standard solution (IV-STOCK-50; Inorganic Ventures, Christiansburg, VA, USA), with single standard solutions for P and S (Inorganic Ventures, USA) added separately to the mixture. Two certified reference materials were used to determine the accuracy of the data: NIST SRM 1573a tomato leaves and NIST SRM 1547 peach leaves (Gaithersburg, MD, USA). Data are given on a dry weight basis and are expressed in g/kg for macroelements and mg/kg for microelements.

### 2.4. Statistical Analyses

Analysis of variance (ANOVA) was computed to test the differences between the (pseudo)cereal grains as well as between their fractions. A principal component analysis (PCA) was performed to determine the parameters that can distinguish between different species and grain fractions. Statistical analyses were conducted using XLSTAT 2014 (Addinsoft, New York, NY, USA).

## 3. Results and Discussion

### 3.1. Variations in Compositional Characteristics

Local grain producers/farmers of various (pseudo)cereals who want to offer minimally processed whole grain products to their end users often face a lack of knowledge and equipment to process small quantities (several dozen to several hundred kg). Whole grains and bran are among the health-promoting ingredients whose consumption should be increased [9]. The technological processes applied to ≈30 kg of harvested, air-dried cereal grains in the present study resulted in a different composition, which formed the basis for distinguishing the cereal fractions. Three wheat fractions (least-, medium- and most-polished grains), four spelt, barley and oat fractions (least-, medium- and most-polished grains and husks) and two common and Tartary buckwheat fractions (most-polished grains and husks) were obtained from the harvested/unpolished grains (Table 2). For barley, oat, common and Tartary buckwheat, the most-polished grain fraction was called groats, while for wheat and spelt, the least- and medium-polished grain fractions were called groats. The suitability of the various fractions for consumption varied by species, as can be seen in Table 2.

(Pseudo)cereal grains require at least some degree of processing to make them edible and palatable and to improve their digestibility [5]. While wheat usually only needs to be cleaned (i.e., winnowing procedure) before consumption, spelt requires an additional threshing step. For barley and oats, husks can be removed by gradually pressing the millstones, which gently brushes/polishes them out. In common and Tartary buckwheat, such a method does not lead to success, so it is necessary to use the method of hulling. Although naked or hulled barley and oat varieties exist, they have disadvantages, such as protrusion of the central radicle above the grain surface, which damages the embryo during threshing, low resistance to drought, plant lodging and various diseases, lower adaptability to changing environmental conditions, etc. [28,29,30,31]. Polishing or slight peeling of the grain to remove the surface layers of the grain may also affect processing performance by removing microbially derived enzymes and hard lignocellulosic structures covering the grain, while leaving most of the dietary fiber and associated compounds in the raw material [9].

Physical parameters of the (pseudo)cereal samples tested included measurements of TKW, kernel width and percent of fraction yield where applicable, as shown in Table 2. TKW and kernel width are expected to decrease with processing. However, few data are available showing the yield of processed grain destined for consumption compared to harvested/unprocessed grain. Here, TKW varied considerably from 13.31 g to 52.24 g. The TKW of the unpolished/least-polished grain decreased between species in the following order: barley (52.24 g) > wheat (51.59 g) > spelt (42.61 g) > common buckwheat (34.71 g) > oat (30.12 g) > Tartary buckwheat (22.74 g). In general, the TKW of all species decreased after the grain was processed. The TKW of barley groats was about 11%, of oat groats, 18%, of buckwheat groats, 31%, and of Tartary buckwheat groats, 41% lower than that of harvested/unpolished grains. In wheat and spelt, both the least- and medium-polished grains were designated as groats. The kernel width ranged from 2.40 mm (oat and Tartary buckwheat groats) to 3.80 mm (harvested/unpolished grain of common buckwheat). There was a tendency for both TKW and kernel width to decrease during the polishing process. The yield of the fractions depended on the grain type, as the husks were not applicable to wheat, and the least- and medium-polished grain fractions were not applicable to either buckwheat type due to the difference in processing. Among the husked grain (pseudo)cereals, the order of husk fraction by frequency was oat (41%) > Tartary buckwheat (40%) > spelt (38%) > common buckwheat (30%) > barley (20%). The total percentage of harvested grains suitable for consumption was highest for wheat (98%), followed by barley (78%), common buckwheat (70%), spelt (62%), Tartary buckwheat (60%) and finally oat (59%). The total proportion of groats in the harvested grains that constituted the final edible product after grain processing was highest for wheat (97%), followed by common buckwheat (70%), Tartary buckwheat (60%), barley (58%), spelt (51%) and oat (34%). The edible fractions of the grains that are not considered groats can be milled into flour. According to the data in the literature, the proportion of barley husks is 10–20% and depends on the variety, growing conditions and processing of the grain [22,32]. The percentage of oat husks is usually higher than barley and is 25–30% of the dry weight of the grain, which is less compared to our results [33]. Significant genetic variation in TKW (16.5–39.8 g) was reported for common buckwheat groats [34]. Jokinen et al. [11] found that the differences between the husk content of laboratory-scale and mill-scale oats were batch dependent, averaging 44.2% and 53.6%, respectively.

Nutritional characteristics included determination of protein, fat and β-glucan content (Table 2). Protein content ranged from 23 mg/g to 149 mg/g and decreased among species in harvested/unpolished grains in the following order: wheat (139 mg/g) > Tartary buckwheat (125 mg/g) > common buckwheat (122 mg/g) > barley (119 mg/g) > oat (112 mg/g) > spelt (108 mg/g). In spelt, the protein content was higher in the least-polished fraction, i.e., in the threshed grains (142 mg/g) rather than in the harvested grains. As for groats of the studied (pseudo)cereals, the highest protein content was found in Tartary buckwheat groats (149 mg/g), followed by spelt groats (142 mg/g), wheat groats (139 mg/g), oat groats (137 mg/g), barley groats (113 mg/g) and common buckwheat groats (105 mg/g). The husks had the lowest protein contents (23–52 mg/g) among the fractions. The latter is expected since the husks are composed of cellulose, hemicellulose and lignin and constitute a significant portion of the unprocessed grains [33]. As described in the introduction, cereal grains usually have a protein content of 7.5–20%, which is in agreement with the results obtained here [2,16]. The fat contents of the studied (pseudo)cereal samples ranged from 3 mg/g to 70 mg/g, with the lowest contents found in the husks. Among the cereals, the highest fat contents were found in oat samples. Fat content of harvested/unpolished grains decreased in the following order: oat (52 mg/g) > Tartary buckwheat (26 mg/g) > barley (22 mg/g) > common buckwheat (21 mg/g) > spelt (19 mg/g) > wheat (16 mg/g). In general, processing harvested grains increased the protein content of most of the studied cereals, except common buckwheat. On the other hand, fat content increased when wheat, oat and Tartary buckwheat were processed, while it decreased in spelt, barley and common buckwheat. The results for protein and fat content are comparable to the data reported by Nogala-Kałucka et al. [35] for hulled and dehulled wheat, barley and oats. Significant genetic variation in protein content (102–236 mg/g) was found in common buckwheat groats, while protein content in husks ranged from 30 to 65 mg/g [34,36].

The β-glucan content varied considerably among the studied (pseudo)cereal samples, ranging from 0.16 mg/g to 54.40 mg/g. There were large differences among species, ranging from the highest β-glucan contents in barley and oat (49 and 34 mg/g, respectively) to intermediate contents in wheat and spelt (9.1 and 6.2 mg/g, respectively) and low contents in common and Tartary buckwheat (0.8 and 0.3 mg/g, respectively) in harvested/unpolished grains. These results are consistent with the higher levels of β-glucans found in oats and barley (32–57 mg/g) and lower levels in wheat and spelt (2–11 mg/g) [4,37]. When husks were compared, the highest β-glucan content was found in spelt husks (2.7 mg/g), followed by oat husks (1.2 mg/g), barley husks (1.0 mg/g) and common and Tartary buckwheat husks (0.5 and 0.3 mg/g, respectively). Brushing/polishing had the greatest effect on oats, where β-glucan content increased by more than 50%. High β-glucan contents (4–8%) in oat and barley grains obtained from bran concentrate have been reported previously [38]. The physical properties of different mill products such as oat groats and flours are mainly related to the chemical composition of the raw material but are also interdependent [11]. Tóth et al. [39] reported that the β-glucan content in threshed spelt grains from 90 genotypes ranged from 4.53 to 8.46 mg/g. Giordano et al. [40] studied wholemeal flours and pearled fractions of barley and common wheat, and the β-glucan contents ranged from 2.2–39.4 mg/g and 6.8–17.6 mg/g, respectively.

A total of 12 elements were determined in the 25 (pseudo)cereal samples, which can be divided into two groups: the macroelements (>0.2 g/kg), K, P, Mg, S and Ca; and the microelements (>0.04 mg/kg), Mn, Fe, Zn, Na, Cu, Cr and Mo. The order of elements from most abundant to least abundant according to the ICP-MS data here was K > P > Mg > S > Ca > Fe > Zn > Mn > Na > Cu > Cr > Mo. As shown in Table 2, the highest levels of element K were found in common buckwheat and oat husks: 5.78 and 5.43 g/kg, respectively. In contrast, Tartary buckwheat and spelt husks contained the lowest levels of this element: 2.59 and 2.14 g/kg, respectively. The range of other macroelements was 0.55–4.85 g/kg for P, 0.39–2.46 g/kg for Mg, 0.29–1.74 g/kg for S, and 0.23–2.24 g/kg for Ca. Here, the concentration of macroelements Mg and P was in the higher range, while the concentration of microelements Fe and Mn was in the lower range than reported in the literature for wheat, barley and oats [41]. The highest relative difference was observed for Ca, with Tartary buckwheat husks having a 10-fold higher Ca concentration than common buckwheat groats. The highest relative differences in microelements were observed for Cr (33-fold) between most-polished wheat grain and Tartary buckwheat husks; for Mo (27-fold) between barley husks and unpolished common buckwheat grains; and for Fe (20-fold) between spelt husks and spelt most-polished grains. The average Zn content in wheat grains worldwide is 28.5 mg/kg, which is slightly higher than in our case [42]. Klepacka et al. [10] found differences between hulled and dehulled common buckwheat grains in terms of Mg, Mn, Zn and Cu content. Dehulled grains contained higher concentration of Mg and Zn, while Mn content had decreased.

### 3.2. Analysis of Variance

Analysis of variance (ANOVA) revealed significant differences among (pseudo)cereal species for 11 of the 18 parameters analyzed. For physical and nutritional characteristics, non-significant differences were detected only for the fraction yield and the protein content, respectively. Regarding the multi-element profile, non-significant differences were found between cereal species for the three macroelements K, P and Ca, and the two microelements Zn and Na. Moreover, ANOVA showed that grain fractions differed significantly for most of the analyzed parameters. Non-significant differences were observed only for some parameters related to the multi-element profile (K, Mn, Fe and Na) (Table 3). Similar to our results, Giordano et al. [40] found significant differences among the fractions of various cereals in several parameters such as protein content and β-glucans. Significant variation in the content of Fe, Zn and Mn were reported for wheat, barley and oat grains from 65 commercial varieties [43]. In common buckwheat, significant differences in Mg, Mn, Zn and Cu content were found in thermal and non-thermal processing of grains [10].

As shown in Table 4, wheat and barley had significantly higher TKW values compared to the other species, while Tartary buckwheat had the lowest values. Kernel width was significantly higher in wheat, spelt, barley and common buckwheat than in oat and Tartary buckwheat. Protein content was significantly higher in wheat, while fat content was higher in oat. β-glucans content was significantly higher in barley and oat. In macroelements, no significant differences were found in K and P content between cereal species, Mg and Ca concentrations were significantly higher in common and Tartary buckwheat and S concentration was significantly higher in wheat and oats. In our study, three edible fractions resulting from brushing/polishing (least-, medium- and most-polished) were limited by two non-edible fractions (raw harvested grains and husks). In general, non-significant differences were found between fractions in most cases, suggesting that brushing/polishing did not greatly affect physical and nutritional characteristics or the multi-element profile. Nevertheless, comparing the unprocessed non-edible fractions with the edible fractions, our results show that the latter have a significant decrease in fractional yield and content of the macroelement Mg compared to the first non-edible fraction (harvested/unpolished grain), while the content of β-glucans increased. However, the other physical parameters, nutritional characteristics and multi-element profile of the harvested grain were not significantly affected until it was highly brushed/polished. A specific pattern was observed in the husks, the non-edible fraction produced during the processing of the grain, compared to other fractions. Thus, the husks are characterized by a very low content of nutritional parameters (protein, fat, β-glucan). Similarly, compared to the other fractions, the husks contained a significantly lower concentration of P, Mg and S, and a significantly higher Ca content. In terms of microelements, the husks had significantly lower levels of Zn, Cu and Mo compared to the other fractions.

**Figure 1:**
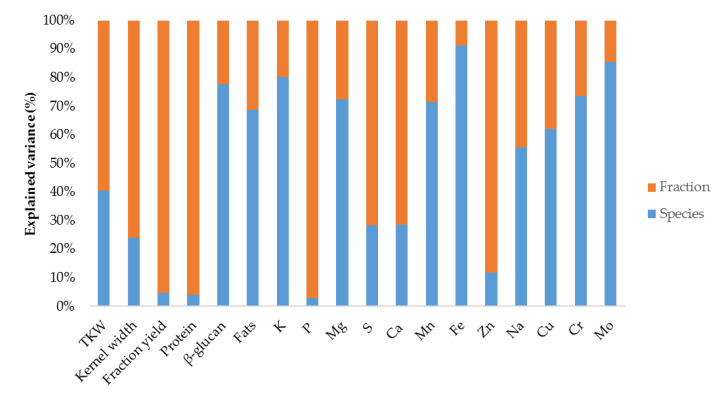
Variance decomposition for the analyzed characteristics.

The variance decomposition resulting from the analysis of ANOVA showed that on average, the inter-specific (between cereal species) and inter-fractions variances accounted for 50.95% and 49.05% of the variance of all parameters, respectively. It is noticeable that with the exception of P (2.92%), Zn (11.71%) and S (28.26%), most of the variance (>50%) of all parameters related to the multi-element profile was due to the differences between cereal species (Figure 1). Regarding the differences between fractions, the greatest variance was observed in the physical parameters of kernel width (75.94%) and fraction yield (95.35%) and in the nutritional parameter “protein content” (96.04), with an average explained variance of 76.92% and 49.88% of the physical and nutritional parameter sets, respectively. In relation to the multi-element profile, the highest variance was observed due to the differences between fractions for P (97.08%) and Zn (88.29%).

### 3.3. Principal Component Analysis

**Figure 2:**
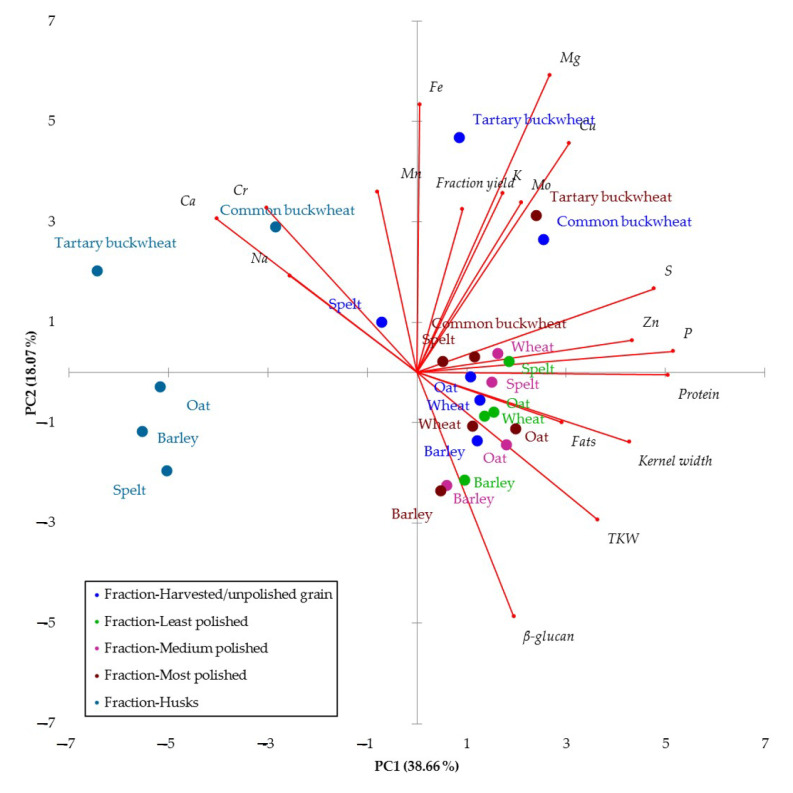
Principal component analysis for the (pseudo)cereal grains based on the data of 18 characteristics.

PCA was performed to identify the parameters responsible for distinguishing (pseudo)cereal types (species) and fractions. The first two principal components, PC1 and PC2, accounted for 38.66% and 18.07% of the total variance, respectively. The 2D plot formed by PC1 and PC2 showed that common buckwheat and Tartary buckwheat appeared to differ from the other cereal grains by high contents of microelements and fraction yield. In agreement with our results, previous studies have shown that buckwheat is characterized by higher content of Mg, K and vitamin B_6_ [44,45] and carbohydrates [46] compared to other (pseudo)cereals. Moreover, Angioloni and Collar [47] showed that buckwheat distinguishes from wheat, spelt, khorasan and rye in terms of mechanical properties (hardness, cohesiveness), dietary fiber (β-glucan), starch hydrolysis parameters and total phenolics. On the other hand, as expected, PCA supported the results of ANOVA, separating the husks from the other fractions originating from grain processing and harvested/unprocessed grain. The husks were characterized by low values of physical and nutritional parameters in all (pseudo)cereal species, indicating that the external/outer layers (i.e., the husks) of the grains have completely different nutritional characteristics and are therefore unsuitable for consumption. However, the husks of common buckwheat appear to have high Cr, Ca and Na content (Figure 2). It has been reported that the surface layers of the physical and chemical properties of the grains can be greatly altered by the gradual polishing, which significantly affects the composition of the fraction and its nutritional value [9,48].

## 4. Conclusions

The compositional characteristics of the studied (pseudo)cereals showed great differences between species and among processed grain fractions. The application of threshing, cleaning and/or brushing/polishing processes to the harvested grains resulted in nutritious, ready-to-eat grain fractions (i.e., groats) suitable for cooking or further processing into flakes or wholemeal flours. Overall, our study suggests that minimum polishing is recommended for wheat and spelt, while maximum polishing for the other species (barley, oat and buckwheat) yields nutrient-rich fractions suitable for direct consumption. As far as the technological processes are concerned, we assume that each (pseudo)cereal species requires specific grain processing that can produce nutrient-rich fractions.

## Figures and Tables

**Table 1:** List of the studied (pseudo)cereals and technological processes applied to the harvested grains.

Latin Name	Species	Variety Name	Processing of Harvested Grains
*Triticum aestivum* L.	Wheat	CCB Ingenio	Winnowing (grain winnowing machine) + brushing/polishing (3×) (adapted traditional stone mill)
*Triticum aestivum* spp. *spelta*	Spelt	Murska bela	Threshing (LD359 machine) + cleaning (Haldrup DC-20) + brushing/polishing (3×) (adapted traditional stone mill)
*Hordeum vulgare* L.	Barley	Concordia	Winnowing (grain winnowing machine) + brushing/polishing (3×) (adapted traditional stone mill)
*Avena sativa* L.	Oat	Noni	Winnowing (grain winnowing machine) + brushing/polishing (6×) (adapted traditional stone mill)
*Fagopyrum esculentum* Moench	Common buckwheat	Eva	Winnowing (grain winnowing machine) + hulling (Sheller device)
*Fagopyrum tataricum* (L.) Gaertn.	Tartary buckwheat	Doris	Winnowing (grain winnowing machine) + hulling (Sheller device)

**Table 2:** Physical and compositional characteristics of brushed/polished (pseudo)cereal grains and husks.

Species	Fraction	Suitable for Consumption	Physical Parameters			Nutritional Characteristics			Multi-Elemental Profile											
			TKW	Kernel Width	FY	Protein	Fats	β-glucan	K	P	Mg	S	Ca	Mn	Fe	Zn	Na	Cu	Cr	Mo
			g	mm	%	mg/g			g/kg					mg/g						
Wheat	Harvested/unpolished grain	Yes	51.59	3.50	100.00	138.94	16.21	9.05	3.61	3.60	1.38	1.45	0.40	31.89	30.28	21.88	8.31	3.45	0.24	0.44
	Least polished	Yes (groats)	50.77	3.40	38.00	139.74	19.63	9.04	3.71	3.60	1.41	1.65	0.39	33.87	30.28	21.10	7.80	3.41	0.14	0.45
	Medium polished	Yes (groats)	49.84	3.40	59.00	139.11	18.55	8.32	4.39	3.85	1.66	1.63	0.44	39.39	36.66	26.42	17.82	4.03	0.14	0.55
	Most polished	Yes	39.81	3.10	1.00	144.89	18.55	8.52	3.75	3.54	1.35	1.64	0.45	32.34	31.57	22.76	7.00	3.38	0.09	0.41
	Husks	n.a.	n.a.	n.a.	n.a.	n.a.	n.a.	n.a.	n.a.	n.a.	n.a.	n.a.	n.a.	n.a.	n.a.	n.a.	n.a.	n.a.	n.a.	n.a.
Spelt	Harvested/unpolished grain	No	n.a.	n.a.	100.00	108.00	18.58	6.15	3.68	3.11	1.26	1.08	0.54	40.22	39.31	22.43	8.56	4.24	0.33	0.96
	Least polished	Yes (groats)	42.61	3.20	51.00	142.25	17.63	8.29	3.91	4.07	1.42	1.27	0.28	37.21	38.65	27.94	8.54	4.88	0.29	0.97
	Medium polished	Yes	32.75	3.00	8.00	138.71	15.15	8.60	3.81	3.82	1.36	1.47	0.31	33.42	36.80	25.66	7.47	4.64	0.19	0.99
	Most polished	Yes	n.a.	n.a.	3.00	134.98	13.96	8.24	3.71	3.53	1.29	1.40	0.33	30.92	46.92	35.92	8.39	4.49	0.29	0.86
	Husks	No	n.a.	n.a.	38.00	24.92	4.53	2.70	2.14	0.66	0.50	0.29	0.64	28.04	20.55	6.44	7.46	1.35	0.61	0.62
Barley	Harvested/unpolished grain	No	52.24	3.60	100.00	118.95	21.94	48.86	4.06	3.54	1.25	1.24	0.53	18.71	35.69	24.83	14.30	4.85	0.23	0.05
	Least polished	Yes	51.40	3.60	5.00	118.59	21.98	48.48	3.95	3.43	1.21	1.14	0.48	15.72	33.90	23.01	12.81	4.65	0.19	0.24
	Medium polished	Yes	49.23	3.57	15.00	116.00	16.86	54.40	3.90	3.34	1.18	0.96	0.39	14.05	31.75	22.79	13.29	4.68	0.54	0.15
	Most polished	Yes (groats)	46.70	3.50	58.00	113.09	17.49	52.58	3.68	3.15	1.10	1.00	0.41	13.49	28.61	20.50	12.07	4.38	0.17	0.07
	Husks	No	n.a.	n.a.	20.00	34.72	5.79	0.96	2.76	0.84	0.84	0.41	1.10	27.39	23.98	10.55	23.11	1.71	0.90	0.04
Oat	Harvested/unpolished grain	No	30.12	2.70	100.00	111.84	51.89	33.62	4.93	3.49	1.04	1.54	0.52	47.28	36.37	24.51	13.76	3.46	0.13	0.49
	Least polished	Yes	27.69	2.60	8.00	129.23	58.84	44.92	4.22	4.03	1.23	1.68	0.61	41.19	34.13	24.77	9.99	4.01	0.41	0.59
	Medium polished	Yes	27.11	2.50	17.00	136.78	67.23	53.03	3.81	3.87	1.15	1.66	0.43	36.74	31.68	24.81	9.56	3.92	0.19	0.55
	Most polished	Yes (groats)	24.56	2.40	34.00	136.84	69.58	50.67	3.98	4.01	1.18	1.69	0.36	36.86	33.26	25.07	9.65	4.00	0.19	0.62
	Husks	No	n.a.	n.a.	41.00	22.69	5.67	1.16	5.43	0.81	0.39	0.75	0.94	34.15	25.14	12.27	24.98	1.22	1.28	0.11
Common buckwheat	Harvested/unpolished grain	No	34.71	3.80	100.00	122.00	21.00	0.82	5.07	3.84	2.36	1.70	0.68	32.96	36.19	20.53	4.42	7.66	0.36	1.08
	Least polished	n.a.	n.a.	n.a.	n.a.	n.a.	n.a.	n.a.	n.a.	n.a.	n.a.	n.a.	n.a.	n.a.	n.a.	n.a.	n.a.	n.a.	n.a.	n.a.
	Medium polished	n.a.	n.a.	n.a.	n.a.	n.a.	n.a.	n.a.	n.a.	n.a.	n.a.	n.a.	n.a.	n.a.	n.a.	n.a.	n.a.	n.a.	n.a.	n.a.
	Most polished	Yes (groats)	23.79	3.30	70.00	105.27	14.13	0.53	3.42	3.07	1.69	1.53	0.23	14.83	35.50	16.88	4.19	6.70	0.22	0.84
	Husks	No	n.a.	n.a.	30.00	52.00	6.00	0.45	5.78	1.29	1.80	0.98	1.28	60.73	37.81	9.77	15.03	6.18	0.33	0.73
Tartary buckwheat	Harvested/unpolished grain	No	22.74	2.90	100.00	125.00	26.00	0.28	4.59	3.83	2.29	1.58	0.89	38.93	414.96	27.47	29.36	6.26	0.84	0.49
	Least polished	n.a.	n.a.	n.a.	n.a.	n.a.	n.a.	n.a.	n.a.	n.a.	n.a.	n.a.	n.a.	n.a.	n.a.	n.a.	n.a.	n.a.	n.a.	n.a.
	Medium polished	n.a.	n.a.	n.a.	n.a.	n.a.	n.a.	n.a.	n.a.	n.a.	n.a.	n.a.	n.a.	n.a.	n.a.	n.a.	n.a.	n.a.	n.a.	n.a.
	Most polished	Yes (groats)	13.31	2.40	60.00	148.48	32.03	0.16	4.95	4.85	2.46	1.74	0.42	21.07	148.31	31.41	8.61	6.80	1.92	0.69
	Husks	No	n.a.	n.a.	40.00	38.00	3.00	0.26	2.59	0.55	1.40	0.45	2.24	38.29	144.17	9.15	17.76	3.67	3.00	0.20

TKW, thousand kernel weight; FY, fraction yield; n.a., not applicable.

**Table 3:** Effect of species and fractions in analyzed characteristics determined by analysis of variance.

	Species		Fraction	
	MSS	*p*	MSS	*p*
TKW	523.836	**0.006**	961.378	**0.000**
Kernel width	1.943	**0.046**	7.665	**0.000**
FY	194.563	0.830	4985.117	**0.000**
Protein	263.033	0.155	7978.262	**<0.0001**
β-glucan	1319.126	**0.000**	473.717	**0.030**
Fats	973.720	**0.000**	553.781	**0.005**
K	1.045	0.246	0.321	0.762
P	0.189	0.362	7.872	**<0.0001**
Mg	0.800	**<0.0001**	0.383	**0.000**
S	0.231	**0.000**	0.733	**<0.0001**
Ca	0.171	0.046	0.535	0.001
Mn	276.429	**0.020**	137.314	0.165
Fe	20687.415	**0.001**	2513.799	0.450
Zn	22.995	0.224	216.684	**<0.0001**
Na	62.018	0.082	62.085	0.092
Cu	8.218	**<0.0001**	6.273	**<0.0001**
Cr	1.131	**0.000**	0.507	**0.015**
Mo	0.410	**<0.0001**	0.087	**0.001**

MSS, mean sum of squares; TKW, thousand kernel weight; FY, fraction yield. Values in bold are significant at *p* ≤ 0.05.

**Table 4:** Means comparison of species and fractions for the analyzed characteristics.

		TKW	Kernel Width	FY	Protein	Fats	β-Glucan	K	P	Mg	S	Ca	Mn	Fe	Zn	Na	Cu	Cr	Mo
		g	mm	%	mg/g			g/kg					mg/kg						
Species	Wheat	48.00 a	3.35 a	49.50 a	140.67 a	18.23 b	8.73 b	3.86 a	3.65 a	1.45 b	1.59 a	0.42 b	34.37 ab	32.20 b	23.04 a	10.23 a	3.57 c	0.15 b	0.46 b
	Spelt	37.68 ab	3.10 a	40.00 a	109.77 b	13.97 b	6.80 b	3.45 a	3.04 a	1.17 bc	1.10 bc	0.42 b	33.96 ab	36.45 b	23.68 a	8.09 a	3.92 c	0.34 b	0.88 a
	Barley	49.89 a	3.57 a	39.60 a	100.27 b	16.81 b	41.06 a	3.67 a	2.86 a	1.12 bc	0.95 c	0.58 b	17.87 b	30.79 b	20.34 a	15.12 a	4.05 c	0.41 b	0.11 c
	Oat	27.37 b	2.55 b	40.00 a	107.48 b	50.64 a	36.68 a	4.47 a	3.24 a	1.00 c	1.46 a	0.57 b	39.24 a	32.12 b	22.29 a	13.59 a	3.32 c	0.44 b	0.47 b
	Common buckwheat	29.25 b	3.55 a	66.67 a	93.09 b	13.71 b	0.60 b	4.76 a	2.73 a	1.95 a	1.40 ab	0.73 ab	36.17 ab	36.50 b	15.73 a	7.88 a	6.84 a	0.30 b	0.89 a
	Tartary buckwheat	18.03 c	2.65 b	66.67 a	103.83 b	20.34 b	0.23 b	4.04 a	3.08 a	2.05 a	1.26 abc	1.18 a	32.76 ab	235.81 a	22.67 a	18.58 a	5.58 b	1.92 a	0.46 b
Fractions	Harvested/unpolished grain	38.28 a	3.30 a	100.00 a	120.79 a	25.94 a	16.46 ab	4.32 a	3.57 a	1.60 a	1.43 a	0.59 b	35.00 a	98.80 a	23.60 a	13.12 a	4.99 a	0.36 b	0.59 a
	Least polished	43.12 a	3.20 a	25.50 b	132.45 a	29.52 a	27.68 a	3.95 a	3.78 a	1.32 ab	1.44 a	0.44 b	32.00 a	34.24 a	24.20 a	9.79 a	4.24 a	0.26 b	0.56 a
	Medium polished	39.73 a	3.12 a	24.75 b	132.65 a	29.45 a	31.09 a	3.98 a	3.72 a	1.34 ab	1.43 a	0.39 b	30.90 a	34.22 a	24.92 a	12.03 a	4.32 a	0.26 b	0.56 a
	Most polished	29.63 b	2.94 b	37.67 b	130.59 a	27.62 a	20.12 ab	3.91 a	3.69 a	1.51 a	1.50 a	0.37 b	24.92 a	54.03 a	25.42 a	8.32 a	4.96 a	0.48 b	0.58 a
	Husks	n.a.	n.a	33.80 b	34.47 b	5.00 b	1.11 b	3.74 a	0.83 b	0.99 b	0.58 b	1.24 a	37.72 a	50.33 a	9.63 b	17.67 a	2.83 b	1.22 a	0.34 b

TKW, thousand kernel weight; FY, fraction yield; n.a., not applicable. Mean values with different letters (a,b,c) in a column are significantly different (*p* ≤ 0.05; differences between species and fractions).

## Data Availability

Data is contained within the article.

## References

[1] Wang J., Chatzidimitriou E., Wood L., Hasanalieva G., Markellou E., Iversen P.O., Seal C., Baranski M., Vigar V., Ernst L. (2020). Effect of wheat species (*Triticum aestivum* vs. *T. spelta*), farming system (organic vs. conventional) and flour type (wholegrain vs. white) on composition of wheat flour—Results of a retail survey in the UK and Germany—2. Antioxidant activity, and phenolic and mineral content. Food Chem. X.

[2] Poutanen K.S., Kårlund A.O., Gómez-Gallego C., Johansson D.P., Scheers N.M., Marklinder I.M., Eriksen A.K., Silventoinen P.C., Nordlund E., Sozer N. (2022). Grains—A major source of sustainable protein for health. Nutr. Rev..

[3] Guo H., Wu H., Sajid A., Li Z. (2021). Whole grain cereals: The potential roles of functional components in human health. Crit. Rev. Food Sci. Nutr..

[4] Biel W., Kazimierska K., Bashutska U. (2020). Nutritional value of wheat, triticale, barley and oat grains. Acta Sci. Pol. Zootech..

[5] Seal C.J., Courtin C.M., Venema K., de Vries J. (2021). Health benefits of whole grain: Effects on dietary carbohydrate quality, the gut microbiome, and consequences of processing. Compr. Rev. Food Sci. Food Saf..

[6] Călinoiu L.F., Vodnar D.C. (2018). Whole grains and phenolic acids: A review on bioactivity, functionality, health benefits and bioavailability. Nutrients.

[7] Tieri M., Ghelfi F., Vitale M., Vetrani C., Marventano S., Lafranconi A., Godos J., Titta L., Gambera A., Alonzo E. (2020). Whole grain consumption and human health: An umbrella review of observational studies. Int. J. Food Sci. Nutr..

[8] Kulathunga J., Reuhs B.L., Zwinger S., Simsek S. (2021). Comparative study on kernel quality and chemical composition of ancient and modern wheat species: Einkorn, emmer, spelt and hard red spring wheat. Foods.

[9] Heiniö R.L., Noort M.W.J., Katina K., Alam S.A., Sozer N., De Kock H.L., Hersleth M., Poutanen K. (2016). Sensory characteristics of wholegrain and bran-rich cereal foods – a review. Trends Food Sci. Technol..

[10] Klepacka J., Najda A., Klimek K. (2020). Effect of buckwheat groats processing on the content and bioaccessibility of selected minerals. Foods.

[11] Jokinen I., Sammalisto S., Silventoinen-Veijalainen P., Sontag-Strohm T., Nordlund E., Holopainen-Mantila U. (2022). Variation in the physical properties of oat groats, flakes and oat flake flour—Processability of thirty pure cultivar oat batches from Finland. LWT—Food Sci. Technol..

[12] Beloshapka A.N., Buff P.R., Fahey G.C., Swanson K.S. (2016). Compositional analysis of whole grains, processed grains, grain co-products, and other carbohydrate sources with applicability to pet animal nutrition. Foods.

[13] Kumar D., Kalita P. (2017). Reducing postharvest losses during storage of grain crops to strengthen food security in developing countries. Foods.

[14] Rathan N.D., Krishna H., Ellur R.K., Sehgal D., Govindan V., Ahlawat A.K., Krishnappa G., Jaiswal J.P., Singh J.B., Sv S. (2022). Genome-wide association study identifies loci and candidate genes for grain micronutrients and quality traits in wheat (*Triticum aestivum* L.). Sci. Rep..

[15] Ibba M.I., Palacios-Rojas N., Rosales-Nolasco A., Rakszegi M., Papageorgiou M., Rocha J.M. (2023). Screening and use of nutritional and healthrelated benefits of the main crops. Developing Sustainable and Health-Promoting Cereals and Pseudocereals: Conventional and Molecular Breeding.

[16] Caporaso N., Whitworth M.B., Fisk I.D. (2018). Protein content prediction in single wheat kernels using hyperspectral imaging. Food Chem..

[17] Wang J., Baranski M., Korkut R., Kalee H.A., Wood L., Bilsborrow P., Janovska D., Leifert A., Winter S., Willson A. (2021). Performance of modern and traditional spelt wheat (*Triticum spelta*) varieties in rain-fed and irrigated, organic and conventional production systems in a semi-arid environment; results from exploratory field experiments in Crete, Greece. Agronomy.

[18] Dolijanović Ž., Nikolić S.R., Subić J., Jovović Z., Oljača J., Bačić J. (2022). Organic spelt production systems: Productive and financial performance in three orographic regions. Ital. J. Agron..

[19] Anders A., Kolankowska E., Choszcz D.J., Konopka S., Kaliniewicz Z. (2020). The effect of selected parameters on spelt dehulling in a wire mesh cylinder. Sustainability.

[20] Takač V., Tóth V., Rakszegi M., Mikó P., Mikić S., Mirosavljević M. (2022). The Influence of farming systems, genotype and their interaction on bioactive compound, protein and starch content of bread and spelt wheat. Foods.

[21] Geisslitz S., Longin C.F.H., Scherf K.A., Koehler P. (2019). Comparative study on gluten protein composition of ancient (einkorn, emmer and spelt) and modern wheat species (durum and common wheat). Foods.

[22] Sharma P., Kotari S.L. (2017). Barley: Impact of processing on physicochemical and thermal properties—A review. Food Rev. Int..

[23] Tömösközi S., Jaksics E., Bugyi Z., Németh R., Schall E., Langó B., Rakszegi M., Rakszegi M., Papageorgiou M., Rocha J.M. (2023). Screening and use of nutritional and health-related benefits of the minor crops. Developing Sustainable and Health Promoting Cereals and Pseudocereals.

[24] Offiah V., Kontogiorgos V., Falade K.O. (2019). Extrusion processing of raw food materials and by-products: A review Vivian. Crit. Rev. Food Sci. Nutr..

[25] Gamel T.H., Rakszegi M., Papageorgiou M., Rocha J.M. (2023). Screening and improving of nutritional and health-related compounds of pseudocereals. Developing Sustainable and Health Promoting Cereals and Pseudocereals.

[26] Luthar Z., Zhou M., Golob A., Germ M. (2021). Breeding buckwheat for increased levels and improved quality of protein. Plants.

[27] Kreft I., Golob A., Vombergar B., Germ M. (2023). Tartary buckwheat grain as a source of bioactive compounds in husked groats. Plants.

[28] Shaveta H.K., Kaur S., Kaur S. (2019). Hulless barley: A new era of research for food purposes. J. Cereal Res..

[29] Lukina K.A., Kovaleva O.N., Loskutov I.G. (2022). Naked barley: Taxonomy, breeding, and prospects of utilization. Vavilov J. Genet. Breed..

[30] Błażewicz J., Kawa-Rygielska J., Leszczyńska D., Grabiński J., Gasiński A. (2021). Influence of variety and nitrogen fertilization on the technological parameters of special malts prepared from naked and hulled oat varieties. Agronomy.

[31] Sterna V., Zute S., Brunava L. (2016). Oat grain composition and its nutrition benefice. Agric. Agric. Sci. Procedia.

[32] Lukinac J., Jukić M. (2022). Barley in the production of cereal-based products. Plants.

[33] Demirel F., Germec M., Coban H.B., Turhan I. (2018). Optimization of dilute acid pretreatment of barley husk and oat husk and determination of their chemical composition. Cellulose.

[34] Lu L., Murphy K., Baik B.K. (2013). Genotypic variation in nutritional composition of buckwheat groats and husks. Cereal Chem..

[35] Nogala-Kałucka M., Kawka A., Dwiecki K., Siger A. (2020). Evaluation of bioactive compounds in cereals. Study of wheat, barley, oat and selected grain products. Acta Sci. Pol. Technol. Aliment..

[36] Gunkova P.I., Buchilina A.S., Ishevskiy A.L., Pchelina E.A., Nikolaev I.A. (2021). Chemical composition of buckwheat groats from various Russian manufacturers. IOP Conf. Ser. Earth Environ. Sci..

[37] Marcotuli I., Houston K., Schwerdt J.G., Waugh R., Fincher G.B., Burton R.A., Blanco A., Gadaleta A. (2016). Genetic diversity and genome wide association study of β-glucan content in tetraploid wheat grains. PLoS ONE.

[38] Yang Z., Xie C., Bao Y., Liu F., Wang H., Wang Y. (2023). Oat: Current state and challenges in plant-based food applications. Trends Food Sci. Technol..

[39] Tóth V., Láng L., Vida G., Mikó P., Rakszegi M. (2022). Characterization of the protein and carbohydrate related quality traits of a large set of spelt wheat genotypes. Foods.

[40] Giordano D., Reyneri A., Locatelli M., Coïsson J.D., Blandino M. (2019). Distribution of bioactive compounds in pearled fractions of tritordeum. Food Chem..

[41] Jordan-Meille L., Holland J.E., McGrath S.P., Glendining M.J., Thomas C.L., Haefele S.M. (2021). The grain mineral composition of barley, oat and wheat on soils with pH and soil phosphorus gradients. Eur. J. Agron..

[42] Wang M., Kong F., Liu R., Fan Q., Zhang X. (2020). Zinc in wheat grain, processing, and food. Front. Nutr..

[43] Loskutov I.G., Khlestkina E.K. (2021). Wheat, barley, and oat breeding for health benefit components in grain. Plants.

[44] Gabrovská D., Fiedlerová V., Holasová M., Mašková E., Smrčinov H., Rysová J., Winterová R., Michalová A., Hutař M. (2002). The nutritional evaluation of underutilized cereals and buckwheat. Food Nutr. Bull..

[45] Kiewlicz J., Rybicka I. (2020). Minerals and their bioavailability in relation to dietary fiber, phytates and tannins from gluten and gluten-free flakes. Food Chem..

[46] Souci S.W., Fachmann W., Kraut H. (2000). Food Composition and Nutrition Tables.

[47] Angioloni A., Collar C. (2011). Nutritional and functional added value of oat, Kamut^®^, spelt, rye and buckwheat versus common wheat in breadmaking. J. Sci. Food Agric..

[48] Abayomi O.O., Gan C.Y., Shafie M.H., Alenezi H., Taiwo A.E., Olumide F.S., Bangar S.P., Kumar M. (2023). Nutritional quality of color cereals and effects of processing on its functional properties. Functionality and Application of Colored Cereals.

